# How Farm Animals React and Perceive Stressful Situations Such As Handling, Restraint, and Transport

**DOI:** 10.3390/ani5040409

**Published:** 2015-12-01

**Authors:** Temple Grandin, Chelsey Shivley

**Affiliations:** Department of Animal Science, Colorado State University, Fort Collins, CO 80523, USA; E-Mail: chelsey.shivley@colostate.edu

**Keywords:** welfare, cattle, sheep, pigs, handling, restraint

## Abstract

**Simple Summary:**

A common animal welfare question is: how stressful is handling and restraining farm animals for veterinary procedures even when no surgical or invasive procedures are done? It depends on how a particular animal perceives it. For one animal, restraint for an injection may be a positive experience associated with food treats and a different animal may be highly fearful and actively resist being restrained. The animal’s response is highly dependent on both its previous experiences and inherited traits such as temperament.

**Abstract:**

An animal that has been carefully acclimated to handling may willingly re-enter a restrainer. Another animal may have an intense agitated behavioral reaction or refuse to re-enter the handling facility. Physiological measures of stress such as cortisol may be very low in the animal that re-enters willingly and higher in animals that actively resist restraint. Carefully acclimating young animals to handling and restraint can help improve both productivity and welfare by reducing fear stress. Some of the topics covered in this review are: How an animal perceives handling and restraint, the detrimental effects of a sudden novel event, descriptions of temperament and aversion tests and the importance of good stockmanship.

## 1. Introduction

There are many situations where farm animals have to be handled, restrained, or transported. Previous reviews and studies have shown that physiological measurements taken after handling and restraint can be highly variable ranging from almost baseline to extremely high [1,2,3]. When some animals are restrained, they react with greater agitated behavior, and higher cortisol, glucose, or lactate, levels compared to others. Restraint for veterinary procedures maybe very stressful for one animal and for another animal it may be relatively low stress. A major factor that is associated with the intensity of either a physiological or behavioral response during handling is previous experience [4,5,6,7]. For a carefully acclimated animal, a restraint device may be associated with food treats and either physiological and behavioral indicators of stress will be low [8,9]. For another animal that has had no previous experience with being restrained it may react violently and physiological stress indicators will be high [2]. Animal welfare scientists are becoming increasingly aware of the importance of both positive and negative affective states [10]. The animal’s affective state may be influenced by its previous experience with handling and restraint. A carefully acclimated animal would probably have a positive affective state and be motivated to enter a restrainer to get a food treat. Another animal that is handled using harsh methods such as shocks would be fearful and have a negative affective state. Aversive handling causes fear and reduces milk production [11].

The stress response is affected by an interaction between the animal’s previous experiences and temperament and other inherited behavioral traits. Procedures for gentling young lambs to get them accustomed to people were more effective for preventing violent reactions during separation from other sheep when they were used on more reactive breeds [12]. More reactive breeds of cattle and sheep may have both higher cortisol levels and more agitated behavior after handling, transport, or restraint [13,14]. The purpose of this review is to summarize how both the animal’s affective state and temperament contribute to differences in behavioral and physiological responses to handling and restraint. Even when no painful surgical procedures are performed, how the animal perceives the experience of being handled and restrained has a major influence on both behavior and physiology [1]. The focus will be on the animal’s reaction to being handled and it will not cover responses to other stressors such as painful surgical procedures, thermal stress or environmental factors. The main emphasis of this review will be on farm animals such as dairy cows, beef cattle, sheep, and pigs. It will also provide suggestions on how to improve an animal’s reaction to handling and restraint.

## 2. Variation between Individuals

### 2.1. Brain Systems Associated with Reactions to Non-Painful Handling or Restraint

Knowledge of the brain systems that have an effect on behavior during handling and restraint can provide insight into how emotional systems have an effect on behavior. In one situation, fear may have a major influence and in another situation, the animal may be reacting to separation distress. The two emotional (affective) systems in the brain that would be most likely to have an effect on both an animal’s physiological or behavioral reaction to non-painful restraint and handling are the fear system and the panic system (separation distress). These subcortical brain systems have been mapped and described [15,16,17,18]. The amygdala is the brain’s fear center. Destruction of the amygdala will have a taming effect on rats and block the animal’s ability to learn a conditioned fear response [19,20]. An example of a conditioned fear response would be an animal that learns to avoid a place where it has received a shock. When the amygdala is stimulated with an electrode, stress hormones such as corticosterone will be secreted [21]. Further evidence that the amygdala is the brain’s fear center is that patients with seizures that start in the amygdala may trigger feelings of dread and activate the autonomic nervous system [22]. These studies clearly show that the brain has circuits that control fearful responses.

Another brain system that may become activated during handling and restraint is the panic or separation distress system [18]. The stria terminalis in the brain is involved with separation distress and can be mediated by brain opioids. This system is activated when the mother and young are separated or a single animal is separated from the herd or flock. When cattle, sheep, or other animals are handled or restrained, they often are separated from other animals. Isolation from herd or flock mates is often highly stressful to both sheep and cattle. Isolated single sheep or cattle may respond with either agitated behavior, increased physiological indicators of stress or increased vocalization [2,23,24,25]. The two brain systems can be affected by genetics. Researchers have bred separate genetic lines of both high and low fear animals and high and low separation distress animals [26]. In quail, researchers were able to clearly show that fear and separation distress are separate traits. Fear and separation stress are controlled by different brain systems [17,18]. The birds were bred to be high or low fear and another group was bred to be either high or low separation distress [27]. They used the term social reinstatement when referring to separation distress. The use of this odd terminology may be due to translation of the original paper from French. Fearfulness was measured with a tonic immobility test and the strength of the social reinstatement trait was measured with a treadmill test. In the tonic immobility test, high fear poultry remain still for a longer period of time. In the treadmill test, the length of time a chick will run against a moving treadmill to stay close to a cage containing its flock mates is measured. Chicks that work harder to stay close, their flock mates have higher separation distress. Therefore inherited factors may have an effect on the strength of the animal’s fear or separation distress.

### 2.2. Breed Temperament, and Age Differences That Have an Effect on Reactions to Handling

A sudden novel event can be highly stressful to an animal [28,29,30]. Inherited differences in fearfulness or reaction to isolation may also affect the intensity of an animal’s reaction [31,32]. There are differences in the reactivity of different breeds of animals. Brahman cross cattle had higher cortisol levels after handling compared to the cattle with no *Bos indicus* genetics [13]. Researchers have also found differences in the reactivity of three different *Bos taurus* breeds of Blond d’Aquitane, Limousin, and Angus [31]. Sheep breed has been shown to affect the intensity of an animal’s reaction to being isolated. Some breeds will vocalize more often than others [32,33]. During many handling procedures animals are temporarily isolated from others when they are restrained for veterinary procedures. A common observation made by many people who show horses or cattle is: “My animal was calm at home, but it became highly agitated at the show.” At a livestock show, there are many new novel stimuli that the animal may have never seen or heard before. A sudden novel stimulus, such as an umbrella opening, a balloon inflating, or being taken to a new place is often used to test genetic differences in fearfulness in many animals [31,34]. Another study with pigs showed that sudden stamping of a boot was one of the most effective tests to determine differences in the reactivity of different breeds of pigs [35]. Younger animals may have a greater reaction than older animals because their responses are less likely to be affected by learning or habituation. An experiment with horses indicated that younger horses had a greater behavioral response to a balloon suddenly inflating compared to older ones [36]. The physiological measurement of heart rate variability showed that even though older horses appeared behaviorally calm, they may have increased nervous system reactivity. For people who are not familiar with heartrate variability, it is the variation in time between successive heartbeats.

Many studies have assessed how inherited factors affect an animal’s reaction to handling and restraint. Researchers [37] found that cattle that were highly reactive to a novel stimulus at the location in which they were reared also had greater physiological responses to handling at a slaughter plant. The animals were most likely responding to the novelty of the slaughter plant environment. Studies have shown significant differences in the responses from different breeds of cattle [38,39]. Beef bulls of different breeds have different stress reactivity responses when tested with a human exposure test and a novel object test. Blond d’Aquitaine bulls were the most reactive, followed by Limousin bulls, and Angus bulls the least reactive [31]. Studies have also shown that cattle that run fast after being released from restraint or become behaviorally agitated and struggle during restraint have lower weight gains [38,39,40]. Cattle with *Bos indicus* breeding usually have higher cortisol levels after restraint [13] and exit from restraint in a squeeze chute more quickly than *Bos taurus* cattle [39,41]. Temperament is a trait that is stable both over time and between situations [42,43]. Individual cattle that became highly agitated in a squeeze chute the first time they were handled continued to be more likely to become agitated when handled in the future [1]. Cattle with less excitable temperaments had higher pregnancy rates, greater weaning weights, better average daily gains, and improved carcass weights compared to cattle with excitable temperaments [44]. To improve productivity, some producers deliberately select beef cattle that remain calm after they have been handled and restrained. Selecting for calm cattle also improves safety for both cattle and humans.

## 3. Methods to Assess Reactions to Handling and How the Animal Perceives Handling and Restraint

There are several different types of behavioral tests that both scientists and producers have used to evaluate animal behavior during routine handling and restraint. Calmer animals are often selected for breeding because they gain weight more easily and are safer to handle [38]. The behaviorial tests described in the next section are, chute score, exit speed scoring, pen scoring, vocalization tests, and aversion tests. The first three tests are often used to assess both inherited differences in temperament and the effects of previous experiences with handling and restraint. Vocalization and aversion test are often used by researchers to assess differences in the aversiveness of different treatments.

### 3.1. Chute Score

The animal’s behavior is scored while it is restrained in a single animal scale, squeeze chute or headgate [45,46]. Observers score each animal on a four or five point scale with scores ranging from stands still, to highly agitated, and struggling. It is likely that this test assesses fearfulness. Highly agitated animals will gain less weight and have poorer meat quality [38,47]. Chute scoring works best when the animal is not tightly restrained by squeeze sides that prevent movement. In studies where the animal was tightly restrained by tight hydraulic sides, exit speed score was more effective for differentiating differences between breeds [40].

### 3.2. Exit Speed Scoring

The speed that a bovine exits from a squeeze chute is electronically measured [39]. Fast exit speeds are associated with lower weight gain and higher physiological indicators of stress [47,48]. Exit speed can also be evaluated with scoring cattle exit gait of, walk, trot, or canter [40,41]. Beef cattle with *Bos Indicus* genetics exited faster than the *Bos taurus* beef breeds [41,49]. It is likely that this test measures differences in cattle fearfulness [50]. Exit speed tests may work poorly with purebred *Bos indicus* cattle such as Nelore, because they may lie down when they become fearful. Purebred *Bos indicus* cattle sometimes freeze instead of having an active response to handling and restraint [51,52].

### 3.3. Pen Scoring

Cattle are held in a small enclosure and a person deliberately invades their flight zone. The test can be used in two ways. It can be used to assess the flight distance or used to determine if the animal will charge and attempt to attack the handler [53]. Animals that remain further away from the tester have a larger flight distance.

### 3.4. Vocalization Tests

There are species differences between sheep, cattle, and pigs in their vocal response to handling or restraint. Several studies have shown that vocalization during handling of cattle and pigs is associated with higher physiological stress measurements [54,55,56]. Cattle will vocalize in direct response to an obviously painful event such as branding [54] or contact with sharp edges or excessive pressure from a restraint device [37,57,58]. When a problem, such as excessive restraint pressure is corrected, the number of cattle that vocalize will greatly decrease [58]. Sheep do not vocalize when they are hurt. Their only defense against predators is flocking and they may remain silent to avoid attracting predators [59]. However, sheep will vocalize loudly when they become separated from other sheep [60,61]. Cattle will also vocalize (moo or bellow) loudly when separated from the herd or when cows and calves are separated [50].

### 3.5. Aversion Tests

Another method to assess the animal’s perception of handling and restraint is to measure its willingness to re-enter a place where it was handled and restrained [9]. This is called an aversion test. Sheep have long memories for negative experiences. A year later they may be reluctant to enter a race where they had been previously restrained [9]. This is an example of one method of aversion testing. The time required to push sheep through a handling race to a device that restrained them became longer with each successive trial [62]. Restraint in an upright position resulted in less aversion with each successive trial compared to clamping and inversion. Shouting and hitting is highly aversive to cows. When a race led to a shouting person, dairy cows moved more slowly through it on the next time they entered [63]. Aversion tests can be used to determine which procedures the animals find most stressful.

Some of the measures that are used during aversion testing are (1) time to move through the single file race that leads to restraint [64]; (2) force required to move an animal through the single file race; or (3) number of refusals to enter the race from a larger holding pen. In a typical force study, the following measures are used to determine the willingness of the animal is to re-enter the place where it was restrained. The animal’s behavior in a single file race leading up to the area of restraint may be classified as (1) no force, walks in when the gate is opened; (2) handler walks past the point of balance by walking back past the animal to urge it to move forward; (3) tap the animal’s rear to move it; or (4) electric prod [41]. Often an animal may turn back and refuse to enter the single file race from a larger holding pen [64]. In some cases, all the behaviors that indicated a reluctance to re-enter, occurred further back in the handling system where the animal perceived that it could avoid being restrained and having a painful procedures. To explain more clearly, the location where refusal behaviors, such as balking, backing up or turning back occurred was in a part of the handling system that was a further distance from the place where a painful treatment occurred. When aversion tests are conducted, reluctance to re-enter must be assessed in both the single file race and in a larger holding pen that leads up to the entrance of the single file race [1].

A second type of test to determine the aversiveness of a handling or restraint method is a choice test. This type of test can be used to determine the relative aversiveness of two different procedures [65]. This test is performed in a Y-shaped race that leads to two different treatments. After the animal has experienced both treatments, it is moved through the Y-race multiple times to determine which treatment it avoids. In one experiment, sheep avoided the side of the Y that led to electrical immobilization and they preferred to be restrained on a restraining table that tilted them to a horizontal position [66].

Another factor that can change aversion testing results is the mildness or the severity of the treatment. Animals will reliably avoid the race that leads to a severe treatment such as electro-immobilization [66]. This type of test indicates how animals perceive different treatments. Severe treatments such as electroimmobilization are perceived as aversive because the animals reliably avoid choosing them when tested in a choice test. However, choice testing may not be reliable for testing choices between mildly aversive treatments [1]. Cattle were reluctant to change a previously learned safe choice when the treatment was only slightly aversive [67]. Cattle were walked through the Y-race and allowed to choose a side. After they had chosen a side and had walked through the chosen path several times they were briefly restrained in the squeeze chute. Most animals continued to go down the previously safe route and did not switch sides. A possible hypothesis for the animal’s tendency to avoid switching to the other side is the fear of the unknown may be greater than the fear of being restrained in the squeeze chute.

In conclusion to this section, methods that measure an animal’s reluctance (aversion) or willingness to re-enter a facility can provide insight into how an animal perceives a procedure. If the animal either refuses or becomes highly reluctant to re-enter, one can conclude that the animal perceives the handling or restraint method as stressful or aversive.

### 3.6. Stress Indicators

#### 3.6.1. Physiological Reactions to Handling and Restraint

Heart rate measurements clearly show that cattle remember painful or aversive procedures. Dairy cows that had experienced electro-immobilization, which feels like an electric shock, had higher heart rates when they returned to the handling facility where electroimmunobilization occurred, compared to cows that had simply been restrained [68].

A basic principle is that when an animal repeatedly and voluntarily re-enters a handling facility or restraint device, physiological measures of stress usually get lower [69]. Bongo antelope that were trained to voluntarily enter a restraint box for blood testing had cortisol levels that were 6.4 ng/mL [8]. Antelopes physically restrained in the wild had levels that were 120 ng/mL [70]. Several studies have shown that cortisol levels will start to decline after cattle or sheep have been handled several times or transported. In both sheep and cattle, an initial trip on a vehicle was more stressful than the seventh or ninth trip [71,72]. This shows that animals acclimate to both handling and transport.

#### 3.6.2. QBA Behavioral Indicators are Associated with Physiological Measures of Stress

Qualitative behavior analysis has become a popular new tool used to assess animal welfare based on body language description. The behavior of sheep was assessed with QBA. Sheep on the initial trip had higher behavioral and physiological indicators of stress. Sheep habituated to transport had significantly lower cortisol [71]. These three studies used Qualitative Behavioral Assessment. QBA is a method where multiple observers blind to treatment use ordinary language to describe behavior [71,72,73]. Sheep that were acclimated to transport were described as comfortable, tired, or confident, and sheep on their first trip were described as alert, anxious or aware. Accordingly, acclimated sheep had lower cortisol levels [62]. Studies where QBA was used to evaluate the long-term stressful effects of housing were less successful [74].

## 4. Ways to Improve Reactions to Handling and How the Animal Perceives Handling and Restraint

Both stock people and researchers will be able to reduce stress during handling and restraint, by being aware of the interaction between inherited temperament differences and the animal’s previous experiences with handling. As stated previously, temperament, tests indicate that inherited traits have an effect on an animal’s reaction to restraint and handling. Cattle that have all been raised and handled on the same farm by the same person have significantly different reactions to temperament tests such as behavioral agitation during restraint or exit speed scoring [38,39,40]. However, these tests do not explain how the animal actually perceives the handling experience. Behavioral tests more accurately assess inherited effects on behavior when they are performed on young animals before they become acclimated and familiar handling and restraint. When no painful procedures are performed, with the exception of vaccination, or blood collection, the temperament scores decreased as animals become calmer with repeated handling and restraint sessions [69]. This probably indicates that the animals are becoming less fearful and more acclimated to handling. Physiological measures also show that animals become acclimated to handling and transport. In sheep, a series of trials where sheep were isolated for one hour showed that cortisol levels decreased with successive trials [61,75].

When an animal is subjected to a painful procedure while held in a restrainer, avoidance behavior may increase and the animal will become more difficult to move through the race [65]. When restraint or veterinary procedures are not painful or only slightly uncomfortable, the animal may acclimate and become increasingly willing to enter the squeeze chute or restrainer [76]. The animal’s reaction to being handled multiple times is determined by both the severity of the procedures, the animal’s previous experience and genetic factors.

### 4.1. Benefits of Acclimating Animals to Handling and Restraint

Various studies have shown the value of acclimating farm animals to handling. Sheep movement through a race was improved by providing barley feed rewards when the sheep exited the system [9]. Three research groups have all reported that walking young calves through the races and handling system created adult animals that were calmer [77,78,79]. More recent studies in the Western U.S. found that acclimating young beef heifers by walking them carefully through the race and squeeze chute produced calmer adult cows. Heifers that had been acclimated, exited more slowly when they were released from the squeeze chute [7]. Acclimation to handling before artificial insemination improved conception rates, lowered temperament scores, reduced, cortisol levels and decreased timing to puberty [44]. It is important to acclimate the young animals. Attempts to acclimate older cows to handling have been less successful [44]. This may be due to previous experiences with aversive handling.

Understanding an animal’s natural behavior can provide clues to their affective state. A new test using a natural behavior in red deer can be used to assess habituation to handling. Red deer calves express stress to their dam via opening their preorbital gland. By measuring the opening of the gland during repeated handling events, researchers were able to identify red deer calves with higher stress levels. They determined their level of habituation to handling, by measuring decreased opening associated with habituation [80]. Finding non-invasive measures of animal stress not only improve the quality of the research (since blood draws can invoke stress regardless of treatment), but it can also improve animal welfare.

Pigs can also benefit from being acclimated to moving through the aisles and having people walk through their fattening pens. Walking fattening pigs in the aisles of the barn or having people walking in their pens produced pigs that were easier to move and handle [81,82,83].

### 4.2. Positive First Experiences are Beneficial for Acclimating Animals to Restraint and Handling

An animal’s first experience with handling and restraint should be positive, as their memories of a negative handling event can last a long time. A study in piglets demonstrated that the response to a human depends on whether their previous experience was positive or negative, and this memory persisted for up to five weeks [4]. This study showed that passive human presence was sufficient to habituate them to humans, though gentle handling was better at habituating them to active human approach. Another study on piglets evaluated the effects of early human handling on their behavior and body weight [84]. Piglets that had been exposed to gentle human handling early in life showed decreased fear reactions in a human approach test, though they did not have increased growth rates. Daily gentle human contact in lambs resulted in less reactive lambs, and they had lower physiological indicators of stress compared to the other treatment groups [85].

### 4.3. Training Animals to Voluntarily Enter Restraint

When nonpainful or slightly painful procedures are performed, animals can be easily trained to re-enter restraint devices. The first author trained Suffolk ewes to voluntarily enter a restrainer that tilted them on their sides. The reward used was a small teaspoon of grain [76]. The ewes were tame and accustomed to people and they quickly learned to run back around through the handling facility and wait at the entrance gate so they could go in the tilt restrainer again. The behavior of these sheep indicated that they were actively seeking the feed reward. When they were released from the tilt table, they immediately ran out and returned to the entrance gate. During this experiment, they were held in a horizontal position in the tilt table for less than a minute and no procedures were conducted. The tilt table had no sharp edges or bars that dug into the animal’s body. When tilted, the ewe laid on a flat metal sheet. The restraint devices used in this experiment was probably less aversive than a restrainer that turns a sheep upside down. Aversion tests could be used to evaluate different sheep restrainers from an animal welfare standpoint.

It is important to use highly palatable rewards that the animals prefer. A study of dairy calves used preference testing to evaluate different feeds or brushing as potential rewards. The calves preferred grain concentrate to normal feed (silage and hay) and they preferred to be brushed by a human compared to entering an empty stall [86].

### 4.4. Animals will not Habituate to Extreme Handling or Painful Procedures

Animals will not habituate or become acclimated to extremely severe treatments [66]. Tying up sheep for 90 to 120 m resulted in extremely high levels of cortisol of 80 to 100 ng/mL [2]. The first author, Temple Grandin, has observed that cattle subjected to prolonged painful treatment, such as having excessive numbers of students palpate them in artificial insemination class, absolutely refused to re-enter the race where this occurred. The aversion behavior occurred far back in the handling system and the cattle refused to re-enter the single file race. In another study, bulls that had been accidentally banged in the head with the headgate of the squeeze chute remembered it and these animals were more likely to balk and refuse to enter a month later [67]. Animals can be trained to tolerate minor pain such as vaccinations or blood sampling but they may become increasingly more averse to entering a handling facility if they are subjected to severe procedures.

Red deer that had been restrained in a drop floor restrainer were more reluctant to re-enter the race that led to it than deer that just ran through it [64]. Time to run through the single file race remained the same. Possibly the time in the single file race remained constant because the deer may have perceived that running fast may have enabled them to escape. The first author, Temple Grandin, has observed that flighty beef cattle that have previously been bitten by dogs in a single file race will often run through the race very quickly the next time they go through it. Possibly they perceive that running fast will help avoid dog bites. People who handle animals need to keep dogs away from the single file race and design restraint devices to be less aversive.

### 4.5. Account for Specificity of Learning

Animal learning is very specific [87]. Taking specificity of learning into account may help explain why an animal becomes agitated in a situation where the stock people would expect it to remain calm. Training a horse to tolerate sudden opening of a blue and white umbrella does not reduce its reaction to a flapping orange tarpaulin [34]. Another example of specificity of learning is that acclimating cattle to a person feeding the animals does not reduce the animal’s response to the first time it moved through the handling facility [6]. In both of these situations, the handling facility and the tarp were probably perceived as novel and new and the umbrella and feeding by people was perceived as safe and familiar. Another study [88] found that taming sheep so they tolerated close proximity to people did not affect their reactions to shearing or being moved through a race.

In the western United States, many beef cattle on extensive rangeland are handled exclusively on horses. For their entire life, they may have never experienced being moved in and out of pens by a person walking on the ground. The first author has observed many situations where cattle that have been moved exclusively by riders on horses have become agitated and dangerous when they were first moved by people on the ground. These cattle were calm and had a small 1 m flight zone when handled by the familiar person on the horse, but the flight zone greatly expanded when they first saw a person walking up to them. The horse and rider was perceived as safe and familiar and the person walking was frightening and new. This can create a dangerous situation for a person in a small pen because the size of the flight zone may exceed the size of the pen. This can result in cattle running over the person in an attempt to get away. In one informal experiment, the first author found that when the person stood beside the horse, the size of the flight zone was intermediate between the person on the horse and the person on the ground.

In another study, cattle demonstrated their specificity of aversion learning [89,90]. Cattle that had previously been accidentally banged on the head with the headgate willingly entered the squeeze portion of the squeeze chute but they stopped at the headgate [89]. The headgate was the aversive part of the squeeze chute that had hurt them in the past. The first author, Temple Grandin, has also observed that cattle which have previously experienced having their heads restrained with nose tongs will violently toss their heads to avoid attachment of the tongs on their nose. They will usually walk down the race willingly and go in the headgate and only react when they see the tongs. They had learned that the squeeze chute and headgate had not hurt them, but the nose tongs were aversive. To prevent these problems, aversive methods of restraint such as nose tongs, should not be used. When the head needs to be restrained, a halter (head collar) is recommended.

Since animals do not have verbal language, they would have to store their memories in a sensory based format such as pictures, specific sounds, or touches. One study showed that horses that were trained to tolerate large children’s toys reacted to them as if they were a novel object when they were presented at a different orientation [90]. Horses trained to tolerate a blue and white umbrella opening will become agitated when a flapping canvas is presented [32]. Animals may be sensory-based thinkers [88]. The author has seen many situations where animals become frightened due to specific associations. One example was a horse that reacted violently to black cowboy hats, but a white cowboy hat evoked no reaction. This particular horse had been badly abused by a person wearing a black cowboy hat. A white cowboy hat had no effect on the horse’s behavior but when he was approached by a person with a black cowboy hat he tensed up and almost reared. When the person with the black hat backed away, the horse calmed down. The specificity of sensory based learning is important because it may help explain why an animal is calm in some situations and becomes highly agitated in another. Training the horse to tolerate umbrellas does not train it to tolerate other things that look totally different. Specificity of learning can also be used to prevent an animal from becoming agitated during handling. The horse that is afraid of black hats is more likely to remain calm if all the riders and handlers stopped wearing black hats. Sensory based memory is an area which needs a lot more research.

### 4.6. The Importance of Good Stockmanship to Improve Both Handling and Welfare

Scientific studies clearly show the benefits of good stockmanship. Animals that are fearful and move away from handlers are less productive [91,92] Young pigs that were subjected to either gentle handling, stroking, or aversive treatment slaps and shocks had significant differences in productivity. Aversively, treated gilts had lower growth rate [93]. Shouting, slapping dairy cows and tail twisting also resulted in lower milk yield compared to soft talking and stroking [94]. There is evidence that dairy cows, calves, pigs, and sheep can learn to recognize individual people. However, when animals are treated roughly by people, an animal may become afraid of all people [95,96,97].

There is also evidence which shows the importance of handler’s attitude towards the animals [93]. People who like animals are examples of positive attitudes. An example of a negative attitude would be a person who thought pigs were stupid and smelly. People with a positive attitude towards the animals they are handling or raising had calmer more productive animals [91,92,98,99,100]. There is also evidence that stress in animals can be reduced by the presence of a familiar stockperson. Sheep remained calmer when they were isolated when a familiar person was with them.

QBA is useful for evaluating the quality of stockmanship. A recent study applied this concept to humans to determine the human-animal relationship between stockperson and dairy calves [101]. Four handler styles were identified: positive interactions, calm/patient, dominating/aggressive, and insecure/nervous. Calves were categorized based on positive or negative mood and high or low arousal. The results showed that the stockperson’s handling style significantly influenced the calves’ arousal and mood. This study highlights the importance of positive human-animal interactions and the necessity of stockperson training and awareness of attitude.

Animal handling at slaughter plants has the potential to be rough; not only does this negatively impact meat quality, but it can compromise animal welfare. A study performed at four slaughter plants in Sweden found that 64% of cattle received handling that was indicative of impaired animal welfare, and 34% or more received actions associated with severely compromised welfare [102]. By identifying problem areas and behaviors, resources can be utilized to provide better training, and increasing third party audits can help reduce the problem. Non-stun religious slaughter presents additional animal welfare challenges, since the animals must remain restrained for 20 s after cutting the neck. A study evaluating different handling techniques in the United Kingdom found that group handling of lambs for non-stun, religious slaughter resulted in significantly lower plasma cortisol and lactate levels compared to individually handled lambs [103]. This reflects the natural behavior of sheep to flock together, and by trying to handle them according to their natural behavior, we can help alleviate some of the stress associated with slaughter.

## 5. Conclusions

The stressfulness of handling and restraint may be partially determined by how the animal perceives it. For one animal, restraint may be a positive experience associated with food rewards and for another animal with different previous experiences being restrained may be highly aversive. Temperament and overall reactivity can also have an impact on interactions between animals and humans. This has been shown in numerous cattle studies. It is important to understand all of the factors that can influence human-animal interactions when designing employee-training programs. Positive human interactions can have significant beneficial effects on animals and can potentially offset the negative experience due to routine procedures such as rectal palpation [92,104]. Gentle physical contact, such as stock people rubbing piglets, simulates play behavior between other animals or maternal behavior such as licking. It is important to determine that the animal perceives these interactions as positive by conducting preference or motivation tests, as seen with brushing in dairy calves [86]. Qualitative behavior assessments can be used to measure the human-animal relationship in both humans and animals, as seen in dairy calves. Transport is often necessary in an animal’s life, so it is important to try to reduce the negative effects it can leave on an animal. More studies to better understand the benefits of good stockmanship on animal productivity and welfare are needed. Continued research to better understand the effects of restraint, handling and transport will give us better strategies to reducing stress of livestock animals.

## References

[1] Grandin T. (1997). Assessment of stress during handling and transport. J. Anim. Sci..

[2] Apple J.K., Minton J.E., Parsons K.M., Unruh J.A. (1993). Influence of repeated restraint and isolation stress and electrolyte administration on pituary adrenal secretions, electrolytes and other blood constituents of sheep. J. Anim. Sci..

[3] Rivaland E.T.A., Charles I.J., Turner A.J., Pompolo S., Tilbrook A.J. (2007). Isolation and restraint stress results in differential activation of cortitrophin releasing hormone and arginine vasopressin neurons in sheep. Neuroscience.

[4] Brajon S., Laforest J.P., Bergeron R., Tallet C., Hotzel M.J., Devillers N. (2015). Persistency of the piglet’s reactivity to the handler following a previous positive or negative experience. Appl. Anim. Behav. Sci..

[5] Petherick J.C., Doogan V.J., Venus B.K., Holroyd R.G., Olsson P. (2009). Quality of handling and holding yard environment and beef cattle temperament, consequences for stress and productivity. Aust. J. Exper. Agric..

[6] Cooke R.F., Arthington J.D., Austin B.R., Yellich J.V. (2009). Effects of acclimation to handling on performance, reproductive and physiological responses of Brahman crossbred heifers. J. Anim. Sci..

[7] Cooke R.F., Ohmart D.W., Cappellozza B.J., Mueller C.J., DelCurto T. (2012). Effects of temperament and acclimation to handling on reproductive performance of *Bos taurus* beef females. J. Anim. Sci..

[8] Phillips M., Grandin T., Graffam W., Irlbeck N.A., Cambre R.C. (1998). Crate conditioning of bongo (*Tragelaphus eurycerus*) for veterinary and husbandry procedures at Denver Zoological Gardens. Zoo Biol..

[9] Hutson G.D. (1985). The influence of barley food rewards on sheep movement through a handling system. Appl. Anim. Behave. Sci..

[10] Boissey A., Lee C. (2014). How assessing relationships between emotions and cognition can improve farm animal welfare. Rev. Sci. Tech..

[11] Rushen J., DePassille A.M.B. (1999). Fear of people by cows and effects on milk yield, behavior and heart rate at milking. Appl. Anim. Behav. Sci..

[12] Caroprese M., Napolitano R., Boivin X., Albenzio M., Annicchiarico G., Sevi A. (2012). Development of affinity to the stockperson in lambs of two breeds. Physiol. Behav..

[13] Zavy M.T., Juniewicz P.E., Phillips W.A., von Tungeln D.L. (1992). Effect of initial restraint, weaning and transport stress on baseline and ACTH stimulated cortisol responses in beef calves of different genotypes. Aust. J. Vet. Res..

[14] Hall S.J.G., Broom D.M., Kiddy G.N.S. (1998). Effect of transportation on the plasma cortisol and packed cell volume in different genotypes of sheep. Small Rumin. Res..

[15] Rogan M.T., LeDoux J.E. (1998). Emotion: Systems, cells, synaptic plasticity. Cell.

[16] LeDoux J.E. (2000). Emotion circuits in the brain. Annu. Rev. Neurosci..

[17] Panksepp J. (1998). Affective Neuroscience: The Foundations of Human and Animal Emotions.

[18] Panksepp J. (2011). The basic emotional circuits of mammalian brains: Do animals have emotional lives?. Neurosci. Biobehav. Rev..

[19] Davis M. (1992). The role of amygdala in fear and memory. Annu. Rev. Neurosci..

[20] Kemble E.D., Blanchard D.C., Blanchard J.L., Takashi R. (1984). Taming in wild rats following medical amygdaloid lesions. Physiol. Behav..

[21] Redgate E.S., Fahringer E.F. (1973). A comparison of pituitary adrenal activity elicited by electrical stimulation of preoptic amygdaloid and hypothalamic sites in the brain. Neuroendrocinology.

[22] Henriksen G.F. (1973). Status epilepticus partials with fear as clinical expression report on a case and ictal EEG recordings. Epilepsia.

[23] Diess V., Temple D., Liyour S., Racine C., Boux J., Terlouw C., Boissy A. (2009). Can emotional reactivity predict stress response at slaughter in sheep?. Appl. Anim. Behav. Sci..

[24] Guesdon V., Ligout S., Delagrange R., Spedding M., Levy F., Lane A.L., Malpaux B., Chaillou E. (2012). Multiple exposures to familiar cospecific withdrawal is a novel robust stress paradigm in ewes. Physiol. Behav..

[25] Curley K.O., Neuendorf D.A., Lewis A.W., Cleere J.J., Welsh T.H., Randel R.D. (2004). Effects of temperament on stress indicators in Brahman heifers. J. Anim. Sci..

[26] Grandin T., Grandin T. (1989). How to improve livestock handling and reduce stress. Improving Animal Welfare: A Practical Approach.

[27] Faure J.M., Mills A.D., Grandin T. (1998). Improving the adaptability of animals by selection. Genetics and the Behavior of Domestic Animals.

[28] Dantzer R., Mormede P. (1983). Stress in farm animals: A need for re-evaluation. J. Anim. Sci..

[29] Stephens D.M., Toner J.N. (1975). Husbandry influence on some physiological parameters of emotional responses in calves. Appl. Anim. Behav. Sci..

[30] Moberg G.P., Wood V.A. (1982). Effect of differential rearing on the behavioral and adrenocortical response of lambs to a novel environment. Appl. Anim. Ethol..

[31] Bourguet C., Deiss V., Boissy A., Terlouw E.C. (2015). Young Blond d’Aquitaine, Angus and Limousin bulls differ in emotional reactivity: Relationships with animal traits, stress reactions at slaughter and *post*-mortem muscle metabolism. Appl. Anim. Behav. Sci..

[32] Boissey A., Bouix J., Orgeur P., Pascal P., Bibe B., LeNeindre P. (2005). Genetic analysis of emotional reactivitiy of sheep: Effects of genotype of the lambs and their dams. Genet. Sel. Evol..

[33] Wolf B.T., McBride S.D., Lewis R.M., Davies M.H., Haresign W. (2008). Estimates of genetic parameters and repeatability of behavioral traits of sheep in an arena test. Appl. Anim. Behav. Sci..

[34] Leiner L., Fendt M. (2011). Behavioral fear and heartrate response in horses after exposure to novel objects: Effects of habituation. Appl. Anim. Behav. Sci..

[35] Lawrence A.B., Terlouw E.M.C., Illius A.D. (1991). Individual differences in behavioral responses of pigs exposed to non-social and social challenge. Appl. Anim. Behav. Sci..

[36] Buragli P., Vitale V., Banti L., Sighieri C. (2014). Effect of aging on behavioral and physiological responses to stressful stimulus in horses. Behavior.

[37] Bourquet C., Deiss V., Tannugi E.C., Terlouw E.M.C. (2011). Behavioral and physiological reactions of cattle in a commercial abattoir and relationships with organizational aspects of the abattoir and animal characteristics. Meat Sci..

[38] Voisinet B.D., Grandin T., Tatum J.D., O’Connor J.J., Struthers J. (1997). Feedlot cattle with calm temperaments have higher average daily gains than cattle with excitable temperaments. J. Anim. Sci..

[39] Burrows H.M., Dillon R.D. (1997). Relationship between temperament and growth in feedlot and commercial carcass traits of *Bos indicus* crossbreds. Aust. J. Exp. Agric..

[40] Vetters M.D.D., Engle T.E., Ahola J.K., Grandin T. (2013). Comparison of flight speed and exit score as measurements of temperament in beef cattle. J. Anim. Sci..

[41] Baszczak J., Grandin T., Bruber S.L., Engle T.E., Plaatter W.J., Schroeder A.L., Laudert S.B., Schroeder A.L., Tatum J.D. (2006). Effects of ractopomine supplementation on behavior of British Continental and Brahman crossbred steers during routine handling. J. Anim. Sci..

[42] Gosling S.D. (2001). From mice to men what can we learn about personality from animal research?. Psychol. Bull..

[43] Reale D., Reader S.M., Sol D., McDougal P.T. (2007). Dingemanse, N.J. Integrating animal temperament into ecology and evolution. Biol. Rev..

[44] Cooke R.F., Bill E. (2014). Kunkle interdisciplinary beef symposium: Temperament and acclimation to human handling influenced growth, health, and reproductive responses in cattle. J. Anim. Sci..

[45] Hall N.L., Buchanan D.S., Anderson V.L., Carli K.R., Berg E.P. (2011). Working chute behavior of feedlot cattle can be an indicator of cattle temperament and beef carcass composition and quality. Meat Sci..

[46] Hoppe S., Brandt H.R., Konig S., Erhardt G., Gauly M. (2010). Temperament traits in beef calf measured under field conditions and relationships on performance. J. Anim. Sci..

[47] King D.A., Schuelhle-Pfeiffer C.E., Rnadel R., Welsh T.H., Oliphint R.A., Baird B.E., Curley K.O., Vann R.C., Hale D.S. (2006). Influence of animal temperament and stress responsiveness on the carcass quality and beef tenderness of feedlot cattle. Meat Sci..

[48] Muller R., von Keyserlinkg M.A.G., Shah M.A., Schwartzkopf-Genswein K.S.B. (2008). Effect of neck injection and handler visibility on behavioral reactivity of beef steers. J. Anim. Sci..

[49] Café L.M., Robinson D.L., Ferguson D.M., McIntyre B.L., Geesink G.H., Greenwood P.L. (2010). Cattle temperament, persistence of assessments, and associations with productivity, efficiency carcass, and meat quality traits. J. Anim. Sci..

[50] Grandin T., Deesing M.J., Grandin T., Deesing M.J. (2014). Genetics and behavior during handling, restraint, and herding. Genetics and the Behavior of Domestic Animals.

[51] Fraser A.F. (1960). Spontaneously occurring tonic immobility in farm animals. Can. J. Comp. Med. Vet. Sci..

[52] Grandin T. (1980). Livestock behavior as related to handling facilities design. Int. J. Stud. Anim. Prob..

[53] Grignard L., Bovin X., Boissy A., LeNeindre P. (2001). Do beef cattle react consistently to different handling situations?. Appl. Anim. Behav. Sci..

[54] Dunn C.S. (1990). Stress reactions of cattle undergoing ritual slaughter using two methods of restraint. Vet. Rec..

[55] Warriss P.D., Brown S., Adams S.J.M., Conett T.R. (1994). Relationship between subjective and objective assessment of stress at slaughter and meat quality in pigs. Meat Sci..

[56] Lay D.C., Friend T.H., Bowers C.L., Grissom K.K., Jenkins O.C. (1992). A comparative physiological and behavioral study of freeze and hot-iron branding using dairy cows. J. Anim. Sci..

[57] Grandin T. (1998). The feasibility of vocalization scoring as an indicator of poor welfare during slaughter. Appl. Anim. Behav. Sci..

[58] Grandin T. (2001). Cattle vocalizations are associated with handling and equipment problems in slaughter Plants. Appl. Anim. Behav. Sci..

[59] Dwyer C.M. (2004). How has the risk of predation shaped the behavioral responses of sheep to fear and distress. Anim. Welf..

[60] Rault J.L., Boissey A., Bouivin X. (2011). Separation distress in artificially reared lambs depends on human presence and number of conspecifics. Appl. Anim. Behav. Sci..

[61] Torres-Hernandez G., Hohenboken W. (1979). An attempt to assess traits of emotionality in crossbred ewes. Appl. Anim. Ethol..

[62] Hutson G.D., Butler M.L. (1978). A self feeding sheep race that works. J. Agric..

[63] Pajor E.A., Rushen J., de Paiselle A.M.B. (2003). Dairy cattle choice of handling treatments in a Y maze. Appl. Anim. Behav. Sci..

[64] Grigor P.N., Goddard P.J., Littlewood C.A. (1998). The relative aversiveness to farmed red deer of transport, physical restraint, human proximity, and social isolation. Appl. Anim. Behav. Sci..

[65] Rushen J. (1986). Aversion of sheep for handling treatments: Paired-choice studies. Appl. Anim. Behav. Sci..

[66] Grandin T., Curtis S.E., Widowski T.M., Thurmon J.C. (1986). Electro-immobilization *versus* mechanical restraint in an avoid-avoid choice test for ewes. J. Anim. Sci..

[67] Grandin T., Odde K.G., Schutz D.N., Behrens L.M. (1994). The reluctance of cattle to change a learned choice may confound preference tests. Appl. Anim. Behav. Sci..

[68] Pascoe P.J. (1986). Humaneness of an electrical immobilization unit for cattle. Anim. J. Vet. Res..

[69] Grandin T., Grandin T. (2014). Handling facilities and restraint in extensively raised range cattle. Livestock Handling and Transport.

[70] Morton D.J., Anderson E., Foggin C.M., Kock M.D., Tiran E.P. (1995). Plasma cortisol as an indicator of stress due to capture and translation in wildlife species. Vet. Rec..

[71] Wickham S.L., Collins T., Barnes A.L., Miller D.W., Beatty D.T., Stockman C., Blache D., Wemelsfelder F., Fleming P.A. (2015). Qualitative behavioral assessment of transport niave and transport habituated sheep. J. Anim. Sci..

[72] Stockman C.A., Collins T., Barnes A.L., Miller D., Wickham S.L., Beatty D.T., Blache D., Wemelsfelder F., Fleming P.A. (2011). Qualitative behavioral assessment and quantitative physiological measurement of cattle niave and habituated to road transport. Anim. Prod. Sci..

[73] Rutherford K.M.D., Donald R.D., Lawrence A.B., Wemelsfelder L.F. (2012). Qualitative behavioral assessment of emotionality in pigs. Appl. Anim. Behav. Sci..

[74] Andreson S.N., Wernelsfelder F., Sandoc P., Forkman B. (2013). The correlation between qualitative behavioral assessments with welfare quality protocol outcomes in on-farm assessments of dairy cattle. Appl. Anim. Behav. Sci..

[75] Roussel S., Hemsworth P.H., Boissy A., Cuvaux-Ponter C. (2004). Effects of repeated stress during pregnancy in ewes on behavioral and physiological responses to stressful events and birth weight of offspring. Appl. Anim. Behav. Sci..

[76] Grandin T. (1989). Voluntary acceptance of restraint by sheep. Appl. Anim. Behav. Sci..

[77] Fordyce G. (1987). Weaner training. Qld. Agric. J..

[78] Binstead M. (1977). Handling cattle. Qld. Agric. J..

[79] Becker B.G., Lobato J.E.P. (1997). Effect of gentle handling on the reactivity of zebu crossbred calves to humans. Appl. Anim. Behav. Sci..

[80] Ceacero F., Landete-Castillejos T., Bartosova J., Garcia A.J., Bartos L., Komarkova M., Gallego L. (2014). Habituating to handling: Factors affecting preorbital gland opening in red deer calves. J. Anim. Sci..

[81] Abbott T.A., Hunger E.J., Guise J.H., Penny R.H.C. (1997). The effect of experience of handling on a pig’s willingness to move. Appl. Anim. Behav. Sci..

[82] Geverink N.A., Appers A., van de Burgwal E., Lambooij E., Biokhuis J.H., Wiegant V.M. (1998). Effects of regular moving and handling on the behavioral and physiological responses of pigs to pre-slaughter treatment and consequences for meat quality. J. Anim. Sci..

[83] Transport Quality Assurance (2010). Transport Quality Assurance Handbook (Version 5).

[84] De oliveira D., da Costa M.J.P., Zupan M., Rehn T., Keeling L.J. (2015). Early human handling in non-weaned piglets: Effects on behaviour and body weight. Appl. Anim. Behav. Sci..

[85] Pascual-Alonso M., Miranda-de la Lama G.C., Aguayo-Ulloa L., Ezquerro L., Villarroel M., Marin R.H., Maria G.A. (2015). Effect of postweaning handling strategies on welfare and productive traits in lambs. J. Appl. Anim. Welf. Sci..

[86] Westerath H.S., Gygax L., Hillmann E. (2014). Are special feed and being brushed judged as positive by calves?. Appl. Anim. Behav. Sci..

[87] Grandin T., Johnson C. (2005). Animals in Translation.

[88] Mateo J.M., Estep D.Q., McMann J.S. (1991). Effects of differential handling on the behavior of domestic ewes. Appl. Anim. Behav. Sci..

[89] Grandin T. (1993). Behavioral agitation during handling of cattle is persistent over time. Appl. Anim. Behav. Sci..

[90] Hanggi E.G., Broken T.D. The Thinking Horse Cognition and Perception. Proceedings of the 51st Annual Convention of the American Association of Equine Practitioners.

[91] Rushen J., De Passille A.M., Grandin T. (2015). The importance of good stockmanship and its benefits to animals. Improving Animal Welfare: A Practical Approach.

[92] Hemsworth P.H., Coleman G.J. (2010). Human Livestock Interactions. The Stockperson and the Productivity and Welfare of Intensively Farmed Animals.

[93] Hemsworth P.H., Barnett J.L. (1991). The effects of aversively handling pigs either individually or in groups on their behavior, growth and corticosteroids. Appl. Anim. Behav. Sci..

[94] Breuer K., Hemsworth P.H., Barnett J.L., Matthews L.R., Coleman G.J. (2000). Behavioral response to humans and productivity of commercial dairy cows. Appl. Anim. Behav. Sci..

[95] Koba Y., Tanida H. (2001). How do miniature pigs discriminate between people? Discrimination between people wearing coveralls of the same colour. Appl. Anim. Behav. Sci..

[96] Tanida H., Nagano Y. (1998). The ability of miniature pigs to discriminate between a strange and their familiar handler. Appl. Anim. Behav. Sci..

[97] Rybarczyk P., Rushen J., de Passille A.M. (2003). Recognition of people by dairy calves using colour of clothing. Appl. Anim. Behav. Sci;.

[98] Destrez A., Coulon M., Deiss V., Delval E., Boissy A., Boivin X. (2013). The valence of the long lasting emotional experiences with various handlers modulates discrimination and generalization of individual humans in sheep. J. Anim. Sci..

[99] Kauppinen T.K., Vesala K.M., Valros A. (2012). Farmer attitude towards improvement of animal welfare is correlated with piglet production paameters. Livestock Prod. Sci..

[100] Waiblinger S., Menke C., and Coleman G. (2002). The relationship between attitudes, personal characteristics and behavior of stock people and subsequent behavior and production of dairy cows. Appl. Anim. Behav. Sci..

[101] Ellingsen K., Coleman G.J., Lund V., Mejdell C.M. (2014). Using qualitative behaviour assessment to explore the link between stock person behavior and dairy calf behavior. Appl. Anim. Behav. Sci..

[102] Hultgren J., Wiberg S., Berg C., Cvek K., Kolstrup C.L. (2014). Cattle behaviours and stockperson action related to impaired animal welfare at Swedish slaughter plants. Appl. Anim. Behav. Sci..

[103] Bates L.S.W., Ford E.A., Brown S.N., Richards G.J., Hadley P.J., Wotton S.B., Knowles T.G. (2014). A comparison of handling methods relevant to the religious slaughter of sheep. Anim. Welfare.

[104] Waiblinger S., Menke C., Korf J., Bueher A. (2004). Previous handling and gentle interactions affect behaviour and heartrate of dayr cows during veterinary procedures. Appl. Anim. Behav. Sci..

